# The effect of embodied learning on students’ learning performance: A meta-analysis

**DOI:** 10.3389/fpsyg.2025.1658797

**Published:** 2025-08-22

**Authors:** Zhiwei Liu, Haode Zuo, Yan Zhao, Yongjing Lu

**Affiliations:** ^1^School of Mathematics, Yangzhou University, Yangzhou, China; ^2^Department of Mathematics, Taizhou University, Taizhou, China

**Keywords:** embodied learning, learning performance, embodied cognition theory, meta analysis, pedagogical design

## Abstract

**Background:**

Embodied learning has attracted considerable attention in recent years. However, there is no academic consensus on whether embodied learning effectively enhances students’ learning performance.

**Objectives:**

This study aims to examine the overall effect of embodied learning on students’ learning performance through a meta-analysis. It also seeks to explore variations based on moderators such as discipline, educational level, experiment period, sample size, region, learning approach, embodied level and type.

**Methods:**

A meta-analysis was conducted on 46 studies (66 effect sizes) published between 2010 and 2025. These studies were analyzed to calculate the overall effect size (Hedges’ g) and explore potential moderating variables.

**Results and Conclusion:**

The results found that: 1) Embodied learning has a moderately positive effect on students’ learning performance (*g* = 0.406, 95%CI [0.264,0.548]), with no significant differences across regions; 2) The effect of embodied learning is greater in the humanities compared to other disciplines (e.g., math); 3) Compared to other educational levels, embodied learning has the greatest impact on high school students’ learning performance; 4) The impact of embodied learning is significantly greater during a one-term experiment period than other periods; 5) Compared with other sample sizes, the embodied intervention group with more than 50 participants has the best effect on their learning performance. 6) Embodied learning in small groups has a greater effect on students’ learning performance than other learning approaches; 7) High-level embodied learning has a more significant effect on students’ learning performance than low-level embodied learning; and 8) Active embodied learning has a greater effect on students’ learning performance than passive embodied learning. These findings provide valuable insights for future practice and research on embodied learning.

## 1 Introduction

How to improve students’ learning performance has been an enduring topic in education. Scholars and educators have consistently sought effective methods to enhance learning performance, leading to the emergence of various innovative instructional approaches. The rise of embodied cognition theory has brought new perspectives to learning methods, paving the way for the development of embodied learning (Shapiro and Stolz, 2019). Unlike traditional cognitive theories, such as information processing theory and symbolic cognition, which focus primarily on the brain’s processing of abstract information and its symbolic representations, embodied cognition theory asserts that cognition is deeply rooted in bodily experiences and physical interactions with the environment (Varela et al., 2016). It challenges the notion that cognition is solely a mental activity and emphasizes the role of the body in shaping our cognitive processes (Nathan, 2021). In contemporary education, embodied learning has significant potential to transform teaching and learning practices (Castro-Alonso et al., 2024). By leveraging multimodal experiences, it fosters deeper understanding, enhances motivation, and promotes active engagement (Zuo and Lin, 2025; Malinverni et al., 2016). Given its increasing prominence, numerous studies have explored its effectiveness across various educational contexts (Mierowsky et al., 2020; Zhang et al., 2024). For example, Lan et al. (2018) found that different types of embodied learning improved primary school students’ listening performance in English as a foreign language. However, some studies have reached the opposite conclusion (Castro-Alonso et al., 2014; De Nooijer et al., 2013; Skulmowski et al., 2016). For example, Shaghaghian et al. (2024) found that university students perceived significantly more physical load in an augmented reality-based embodied learning environment. Therefore, this study aims to comprehensively assess the effect of embodied learning on students’ learning performance through a meta-analysis. Meta-analysis integrates raw data from numerous empirical studies, allowing researchers to quantify the effect size of interventions (Borenstein et al., 2021). By using this method, researchers can draw stronger and more persuasive conclusions about the effect size of interventions (Lipsey and Wilson, 2001).

Currently, while several meta-analyses have explored the impact of embodied learning on students’ learning performance, most of them seem to focus on the field of educational technology. This represents a significant gap, as the effects of embodied learning across various academic fields remain underexplored. For instance, Yu and Yu (2023) conducted a meta-analysis of 37 studies published between 2010 and 2022, revealing the positive effects of technology-based embodied learning on students learning. Similarly, Zhang et al. (2025) synthesized the results of 44 empirical studies published from 2012 to 2023, demonstrating that technology-based embodied learning is an effective method for enhancing learning effectiveness. However, it is worth noting that some meta-analyses have a limited scope, involving relatively small sample sizes. For example, Lyu and Deng (2024) performed a meta-analysis of 17 studies from a cognitive load theory perspective, examining the positive impact of embodied learning on learning performance. Despite the valuable insights provided by these analyses, they overlook a comprehensive range of moderators, such as embodied level and type. Furthermore, these analyses treated multiple outcomes from the same participants as independent results, which may lead to inflated effect sizes (Tharumalingam et al., 2025; Deng et al., 2024; Liu et al., 2025). In other words, this approach risks overestimating the overall effect of embodied learning on students’ learning performance. Overall, while existing meta-analysis offer evidence regarding the impact of embodied learning on learning performance, their limitations—such as narrow disciplinary focus, small sample sizes, inflated effect sizes, and insufficient exploration of moderators—underscore the need for a more comprehensive integration of empirical evidence.

Therefore, this study aims to address these limitations by systematically investigating the effect of embodied learning across diverse academic fields and thoroughly examining relevant moderators. This study also explores potential moderators, including discipline, educational level, experiment period, sample size, region, learning approach, embodied level, and embodied type. Accordingly, the study addresses the following research questions:

RQ1: Is embodied learning effective in enhancing students’ learning performance?

RQ2: Does the effect of embodied learning on students’ learning performance vary across disciplines, educational levels, experimental periods, sample sizes, regions, learning approaches, embodied levels and types?

## 2 Literature review

### 2.1 Embodied learning

Embodied learning is a method that emphasizes the interaction between the environment, the body, and artifacts during the learning process (Borghi and Cimatti, 2010). It stems from embodied cognition theory, which posits that cognitive processes are deeply rooted in the interactions between the body and the environment (Shvarts and Abrahamson, 2023). In education, learners often enhance their performance by integrating sensory experiences and physical activity into the learning process (Kosmas and Zaphiris, 2023; Andolfi et al., 2017). Embodied learning caters to various learning styles, particularly benefiting students who prefer kinesthetic learning (Post et al., 2013). Furthermore, the introduction of emerging technologies, such as virtual reality (VR) and augmented reality (AR), has expanded the possibilities for embodied learning (Lin et al., 2024). These technologies create immersive learning environments that allow students to engage in physical interactions within virtual worlds, enhancing immersion and facilitating the understanding of abstract concepts (Lui et al., 2020). Similarly, the use of learning tools and manipulatives promotes more interactive and participatory learning through tactile and kinesthetic methods (Ginns and King, 2021). Overall, by leveraging the interconnection between the body, mind, and environment, embodied learning not only affects students’ learning performance but also fosters a more comprehensive and inclusive learning experience (Zhong et al., 2023).

### 2.2 Learning performance

To comprehensively and scientifically assess the effects of embodied learning on students’ learning, this study adopts the term learning performance to measure the embodied learning outcomes and achievements achieved by students (Lyu and Deng, 2024; Yu and Yu, 2023). In education, learning performance is a multifaceted and dynamic concept that encompasses various aspects of students’ performance in academic activities, including academic grades, critical thinking, problem-solving skills, and creativity (Wu et al., 2023). In this study, learning performance serves as a comprehensive assessment criterion to evaluate students’ overall embodied learning outcomes. The assessment covers multiple dimensions and is quantified through measures such as exam scores, paper performance, class participation, project quality, and others (Khan and Ghosh, 2021; Pan et al., 2021).

### 2.3 The effects of embodied learning on students’ learning performance

Studies on embodied learning have shown its positive impact on students’ learning performance across various learning areas (Hong et al., 2024; Hung and Chen, 2018; Macken and Ginns, 2014). For instance, Kwon et al. (2025) found that embodied learning activities, implemented in both mixed-reality and unplugged contexts, significantly enhanced primary school students’ computational thinking. Chettaoui et al. (2022) also revealed that a multimodal embodied learning interface had potential educational benefits for primary school students’ learning performance. However, some studies have found that embodied learning had no significant effect on students’ learning performance (Ginns et al., 2016). For example, Schmidt et al. (2019) argued that while embodied learning was more effective in helping primary school students learn new words than the control group, there was no significant difference in the level of focused attention between the two groups. Similarly, Chang et al. (2025) showed no significant difference in the reflective writing performance between the group using a ChatGPT-integrated embodied learning method and the control group using traditional reflective writing. Therefore, the effect of embodied learning on students’ learning performance remains inconclusive. Based on these conflicting findings, we hypothesize that:

*H1:* Embodied learning has a significant positive effect on students’ learning performance.

### 2.4 Disciplines

Disciplines refer to the academic subjects that learner study through embodied learning. Based on Zhang et al. (2025), it can be categorized into science, humanities, computing, physical education, and others. Science often enhances the embodied experience of abstract concepts through experimental operations and tool usage (Agostinho et al., 2015), while the humanities rely on embodied simulation and contextual experiences to facilitate text comprehension and critical discussion (Chen and Liu, 2025). In physical education, students directly experience motor skills, which deepens their understanding of the underlying principles (Hsiao and Chen, 2016). In the field of computing, students can enhance their understanding of data structures through physical interactions with virtual simulations (Darejeh et al., 2022). Skulmowski and Xu (2022) found that different disciplines may require varying types of cognitive effort, which could influence the effectiveness of embodied learning. Lyu and Deng (2024) also showed that embodied learning was more effective in physics discipline than other disciplines. Therefore, we propose that:

*H2:* Discipline is a significant moderator in the impact of embodied learning on students’ learning performance.

### 2.5 Educational levels

Educational levels refer to the stages of education where embodied learning is applied. These stages can be categorized into pre-school, primary school, middle school, high school, and university, as outlined in Zhang et al. (2025). Existing literature suggests that the effects of embodied intervention may be influenced by individual cognitive development stages (Zou et al., 2025, p.5). For example, Lyu and Deng (2024) found that applying embodied learning to primary students may have better effectiveness. However, Yu and Yu (2023, p.17) noted that the technology-based embodied learning has a large effect on secondary school students. Based on these findings, we propose that:

*H3:* Educational level is a significant moderator in the effect of embodied learning on students’ learning performance.

### 2.6 Experiment periods

Experiment period refers to the period of students’ participation in the experiment (Yu and Yu, 2023, p.8). According to Zhang et al. (2025), it can be categorized into approximately 1 term, 1 month, 1 week, or 1 h. Existing research has demonstrated that embodied learning significantly impacts students’ learning performance across various experimental periods (Smyth et al., 2021). For instance, Cherdieu et al. (2017) found that combining gestures with sufficient learning time facilitated better knowledge integration, particularly in the consolidation of long-term memory. Similarly, Kosmas et al. (2019) argued that the long-term experiment period should be paid to examine the true effectiveness of embodied learning. However, Korbach et al. (2020) suggested that gestures, such as pointing and tracing, positively impacted student learning in the short term. Hu et al. (2015) also demonstrated that tracing elements of geometry worked examples with the index finger enhanced learning outcomes during the short term. Given the inconsistencies in these findings, we hypothesize that:

*H4:* Experiment period is a significant moderator in the impact of embodied learning on students’ learning performance.

### 2.7 Sample sizes

Sample size refers to the number of learners who participated in the embodied intervention group and can be categorized as 0–30, 31–50, and >50 (Yu and Yu, 2023). Sample size has a significant impact on students learning (Breton, 2014). For example, Kuo et al. (2014) found that when sample size is beyond manageable, teachers were unable to attend to all individual students, thereby reducing the effectiveness of physical response learning. Georgiou and Ioannou (2021) also reported that when sample size exceeded manageable limits, students might feel bored while waiting for their turn in technology-enhanced embodied learning. Similar, Huang et al. (2022) revealed that a large sample size increased teachers’ physiological and psychological stress, which led to less personalized guidance for students. Therefore, we propose that:

*H5:* Sample size is a significant moderator in the effect of embodied learning on students’ learning performance.

### 2.8 Regions

Region refers to the location where embodied learning is implemented. According to the study by (Yu and Yu, 2023), it can be divided into Africa, Asia, Australia, Europe, and North America. Existing research has found that regional differences lead to disparities in educational resources and cultural backgrounds, which in turn affect the effectiveness of embodied learning (Lyu and Deng, 2024). Specifically, differences in educational resources (e.g., educational systems, teaching practices) influence students’ receptiveness to various teaching methods and their learning experiences (DeCapua, 2016). In regions with abundant educational resources, where students are exposed to diverse teaching approaches (e.g., animations with gestures) at an early age, they are more likely to adapt to embodied learning (Lajevardi et al., 2017). Furthermore, as embodied learning is influenced by socio-cultural trends, cultural differences significantly shape students’ learning styles (Jusslin et al., 2022; Kolb and Kolb, 2009). Therefore, we hypothesize:

*H6:* Region is a significant moderator in the impact of embodied learning on students’ learning performance.

### 2.9 Learning approaches

Small group, individual, and mixed learning are the three primary approaches in an embodied learning environment (Yu and Yu, 2023). Among these approaches, more researchers tend to focus on group interactions (Jones et al., 2022; Lemke, 2000). For example, Danish et al. (2020) found that collective learning not only shaped individuals’ embodied experiences but also yield additional learning benefits. Liyanawatta et al. (2022) also found that combining drama-based embodied learning activities with collaborative learning in the classroom effectively enhanced high school students’ engagement. Similarly, Johnson-Glenberg et al. (2014b) reported that collaborative embodied learning significantly improved high students’ learning performance compared to traditional learning methods. Therefore, group learning enhances students’ cognitive development and fosters positive attitudes and higher motivation than individual learning (O’Donnell and O’Kelly, 1994). We hypothesize that:

*H7:* Learning approach is a significant moderator in the impact of embodied learning on students’ learning performance.

### 2.10 Embodied levels

Embodied level refers to the degree of physical involvement in embodied activities (Johnson-Glenberg and Megowan-Romanowicz, 2017). According to existing literature (Yu and Yu, 2023), embodied learning can be classified into three levels: low (e.g., mouse clicks or finger movements), middle (partial body movement, e.g., upper body), and high (e.g., whole-body movement). Previous studies have suggested that the degree of body movements can influence learning outcomes (Georgiou et al., 2019; Johnson-Glenberg et al., 2020). For example, some studies found that high-level embodied learning were more beneficial for students’ performance than low-level embodied learning (Conley et al., 2020; Guo and Goh, 2015). However, Mayer and DaPra (2012) argued that compared to low-level embodied learning, high-level embodied learning did not significantly affect university students’ transfer test performance. Given these conflicting findings, we propose that:

*H8:* Embodied level is a significant moderator in the effect of embodied learning on students’ learning performance.

### 2.11 Embodied types

Embodied type refers to the ways where students engage their bodies in the learning process, and it can be categorized into two types (Xu and Ke, 2020). One type is passive embodied learning, where a learner merely observes a remote instructor (either humans or animated figures), or uses the mouse to move someone or something on the screen (Xu et al., 2022). The other type is active embodied learning, where learners manipulate their own bodily movements (Johnson-Glenberg et al., 2014a). Prior research has found that active embodied learning outperforms passive embodied learning in terms of learning outcomes (Hung et al., 2014). For instance, Lindgren et al. (2016) showed that middle school students who used their whole bodies to experience physical concepts learned more effectively than those using a traditional mouse-based interface. However, some studies have suggested that active embodied learning is not always superior (Lan et al., 2018; Xu and Ke, 2023). Given these inconsistencies, we hypothesize that:

*H9:* Embodied type is a significant moderator in the effect of embodied learning on students’ learning performance.

## 3 Method

### 3.1 Data sources and searching strategy

To ensure comprehensive inclusion of relevant literature, this study searched databases such as Web of Science, ProQuest, Wiley Online Library, PubMed, APA PsycNet, ScienceDirect, ERIC, and Scopus, with Google Scholar used as a supplement for additional sources. A citation tracking method was employed for snowball sampling of the collected literature, which included influential educational psychology journals like the *Educational Psychology Review*. The literature search was conducted on June 24, 2025, and only studies published after 2010 were included in this meta-analysis.

Furthermore, the search terms were divided into two groups: the first group included terms related to embodied learning, such as embodied, body, gesture, hand movement, pedagogical agent, physical activity, and embodied cognition; the second group included terms related to the research context, such as education, student, and learner. The study initially connected terms within each group using the Boolean operator “OR” and then linked the two groups with the operator “AND.” During the database search, all references were imported into the EndNote software to prevent duplicates from different databases.

### 3.2 Inclusion and exclusion criteria

To ensure the rigor of this meta-analysis, the following inclusion and exclusion criteria were established. (1) Studies examining the impact of embodied learning on students’ learning performance were included. Studies focusing on outcomes other than learning performance were excluded. (2) Studies comparing embodied learning with other learning methods were included. In studies with multiple experimental groups, only data from the groups using embodied learning interventions exclusively were extracted for analysis. (3) Studies providing quantitative data (means, standard deviations, and sample sizes) from (quasi-)experimental studies were included. Studies with research designs other than (quasi-)experimental, or those lacking full texts and data, were excluded. Furthermore, if delayed tests were conducted, data were extracted only from the first test point. If both subgroup and overall data were reported, the overall data were used. (4) Studies that met the quality assessment standards established in this paper were included, while those that did not meet the standards were excluded. (5) Studies published in English journals and conferences before June 2025 were included, while studies published in other languages were excluded.

**Figure 1:**
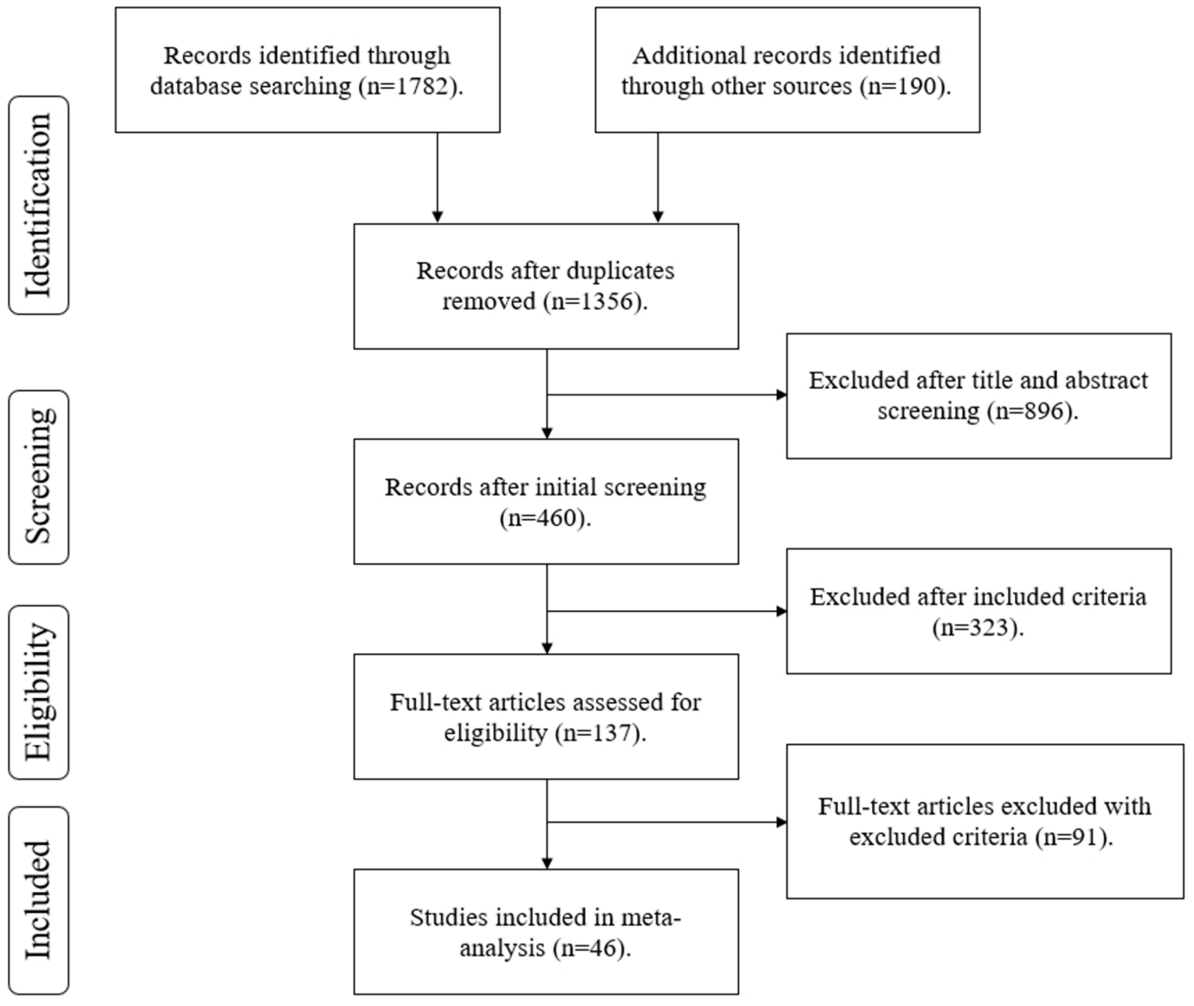
Flow diagram.

Figure 1 shows the process of literature collection, screening, and inclusion. The initial search yielded 1,972 records from multiple databases. After removing 616 duplicate studies, 1,356 studies remained for screening. During the abstract and title screening phase, 896 studies were excluded, leaving 460 studies for full-text assessment. Based on the inclusion criteria, 137 studies were deemed eligible. Subsequently, 91 studies were excluded according to the exclusion criteria, resulting in 46 studies included in the data coding process.

### 3.3 Coding

Based on the coding scheme proposed by Cooper (2016), this study coded the following information from 46 articles: (1) author information, (2) publication year, (3) statistical results of learning performance (e.g., mean, standard deviation), (4) discipline, (5) educational level; (6) experiment period, (7) sample size; (8) region, (9) learning approach, (10) embodied level, and (11) embodied type. Coding details are provided in the Supplementary Appendix.

As shown in Table 1, the selected moderators were categorized based on existing studies. In line with the framework established by Zhang et al. (2025), the moderators were classified as follows: disciplines (science, humanities, computers, physical education, and others), educational levels (university, high school, middle school, primary school, and pre-school), and experiment periods (approximately 1 term, 1 month, 1 week, or 1 h). Furthermore, following the moderator classification criteria proposed by Yu and Yu (2023), the moderators were categorized as follows: sample sizes (0–30, 31–50, and >50), regions (Africa, Asia, Australia, Europe, and North America), learning approaches (small group, individual, and mixed learning), and embodied levels (high, middle, and low). Finally, according to Xu and Ke (2020), embodied learning can be categorized into active or passive embodiment. Two authors independently screened and coded the studies, achieving a consistency of 0.896. Any discrepancies were resolved by consulting the original articles.

**Table 1:** Moderators and their categories.

Moderators	Categories	References
Disciplines	Science (e.g., science, biology, physics, math), humanities (e.g., languages, music, geography), computers, physical education (sports), and others.	Zhang et al. (2025)
Educational levels	University, high school, middle school, primary school, and pre-school.	
Experiment periods	1 term or so, 1 month or so, 1 week or so, and 1 h or so.	
Sample sizes	0–30, 31–50, and >50.	Yu and Yu (2023)
Regions	Africa, Asia, Australia, Europe, and North America.	
Learning approaches	Small group, individual, and mixed.	
Embodied levels	High, middle, and low.	
Embodied types	Active or passive.	Xu and Ke (2020)

### 3.4 Research quality assessment

According to the Standard Quality Assessment Criteria developed by Kmet et al. (2004), the quality of the 46 included studies was assessed. This versatile evaluates all sections of empirical studies (e.g., study design, methods) and has been widely applied in educational research (e.g., Iuga and David, 2024; Fricke et al., 2022). Each study was evaluated across 14 dimensions, with each item scored as yes (2), partial (1), and no (0). The total score for each study was calculated by dividing the sum of its score by 28. According to thresholds established by Kmet et al. (2004), scores of <0.50, 0.50–0.70, 0.71–0.80, and >0.80 indicate low, adequate, good, and high quality, respectively. In this meta-analysis, the quality scores of the 46 studies ranged from 0.72 to 0.94, indicating that all studies met the quality standards required for meta-analysis. The first and third authors independently evaluated each study’s quality, with an inter-rater consistency coefficient of 0.86, indicating a high level of consistency (McHugh, 2012). Any discrepancies in evaluations were resolved by consulting the corresponding author.

### 3.5 Data analysis

To comprehensively explore the effect of embodied learning on students’ learning performance, this study followed the guidelines of the Preferred Reporting Items for Systematic reviews and Meta-Analyses (PRISMA) statement (Page et al., 2021). Comprehensive Meta-Analysis (CMA 3.0) software was used to conduct effect size calculation, heterogeneity test, moderator analysis, publication bias test, and sensitivity analysis on the coded results. The following sections describe these processes.

#### 3.5.1 Calculating effect size

Effect size is a crucial metric for assessing the strength of correlation between variables or the level of experimental effects (Hedges and Vevea, 1998). Given the relatively small sample sizes and the different (quasi-)experimental designs of these studies, Hedges’ g was chosen for calculating effect sizes due to its ability to correct small sample bias and reduce bias in standardized mean differences (Ren et al., 2024). Unlike Cohen’s d, which may overestimate the effect size in small or unequal samples, Hedges’ g offers a more conservative and unbiased estimate, making it suitable for educational studies with different designs and sample sizes. According to Thalheimer and Cook’s (2002) study, an effect size between 0.15–0.40, 0.40–0.75, and >0.75 is interpreted as small, moderate, and large, respectively.

It is important to note that different results from the same participants should not be treated as independent outcomes, as this may lead to a misestimation of the overall effect (Hedges et al., 2010). Various methods have been developed to address this inflation effect. One approach involves extracting a single effect size from multiple comparisons within a single study, using “the study as the unit of analysis” and “subgroups within the study” options in CMA 3.0 to calculate the combined effect size (Çalik et al., 2023, 2024). This approach minimizes the number of individual studies to address the inflation effect caused by multiple experimental outcomes (Rahman and Lewis, 2020).

#### 3.5.2 Heterogeneity test and moderate analysis

To choose between the random-effects and the fixed-effects model, the researchers considered how the characteristics of the included papers matched the prerequisites of each model. The selection of a meta-analysis model depends on assumptions about the distribution of true effect sizes across studies and the intended scope of inference (Borenstein, 2019). The fixed-effects model assumes that all studies share a common true effect size and that observed differences among studies are due to sampling error. According to Hedges and Vevea (1998), the fixed-effects model allows for inferences about the relevant parameters within the analyzed studies and is suitable when the studies are functionally identical, estimating the effect size for a single population. In contrast, the random-effects model assumes that true effect sizes may vary across studies due to moderators (e.g., sample size). It is used when studies are functionally different and can estimate and generalize the effect size to a broader population.

Building on these theoretical foundations, the researchers also examined heterogeneity by calculating the Q-value and I^2^ statistic. The heterogeneity criteria established by Higgins et al. (2003) guided model selection. A significant Q-test indicates the presence of heterogeneity, where the variability in effect sizes exceeds what would be expected due to sampling errors. The I^2^ statistic reflects the proportion of total variance in effect sizes due to heterogeneity, with I^2^ values of 25, 50, and 75% corresponding to low, medium, and high levels, respectively.

Overall, significant heterogeneity suggests that the effect of embodied learning on students’ learning performance may be influenced by moderators. Therefore, further Q-tests across different moderators are necessary to assess whether significant differences in effect sizes exist.

#### 3.5.3 Publication bias and sensitivity analysis

Publication bias refers to the tendency for studies with statistically significant results or large effect sizes to be more likely published than those with non-significant results or smaller effect sizes (Borenstein et al., 2021). To assess publication bias, a funnel plot was used based on the distribution of effect sizes. However, this method relies on qualitative visual inspection, which introduces some subjectivity. Therefore, to achieve symmetry in the funnel plot, the trim-and-fill method was employed to estimate the number of missing studies.

To further quantify the effect of publication bias, Orwin’s fail-safe N was applied to evaluate the number of missing studies required to reduce the effect size to a negligible level (*g* < 0.01). Additionally, the Classic fail-safe N test was used to assess whether publication bias could undermine the overall conclusions (Littell et al., 2008). This method estimates the minimum number of missing studies needed to nullify the observed effect size. According to Rosenthal’s (1995) criterion, the threshold is set at 5n + 10, where n represents the number of studies included. A fail-safe N larger than this threshold suggests that the effect of unpublished studies on the overall effect is likely minimal. Publication bias was also assessed using the Egger’s test, with *p* < 0.05 indicating significant publication bias (Deeks et al., 2005).

Finally, sensitivity analyses were conducted to identify outliers influencing the overall effect (Reitsma et al., 2005). A single-study removal method was employed to assess the impact of studies with extreme negative or positive effects. Studies with small sample sizes that caused large heterogeneity were excluded to improve result stability.

## 4 Results

### 4.1 Descriptive information

Of the 46 articles (66 effect sizes) included in the final analysis, the experimental group consisted of 3,594 participants, while the control group included 3,827 participants. The range of effect sizes was [−0.825, 4.810], with 17 being negative and 49 being positive.

### 4.2 Heterogeneity test

Table 2 presents the results of the heterogeneity test. The Q-value for the overall effect was 536.483 (df = 65, *p* < 0.001), indicating heterogeneity among the samples. The I^2^ value was 87.884%, which is greater than 75%, showing a high degree of heterogeneity. Therefore, this study employed the random-effects model to estimate effect size and used moderator analysis to explore the sources of heterogeneity.

**Table 2:** Overall effect size.

Model	Effect size	95%CI	Heterogeneity test				Tau-squared		
			Q	df	*p*	I^2^	Tau^2^	Tau	SE
Fixed	0.314	[0.267,0.361]	536.483	65	0.000	87.884%	0.288	0.536	0.081
Random	0.406	[0.264,0.548]							

CI, confidence interval.

### 4.3 Overall effectiveness

**Figure 2:**
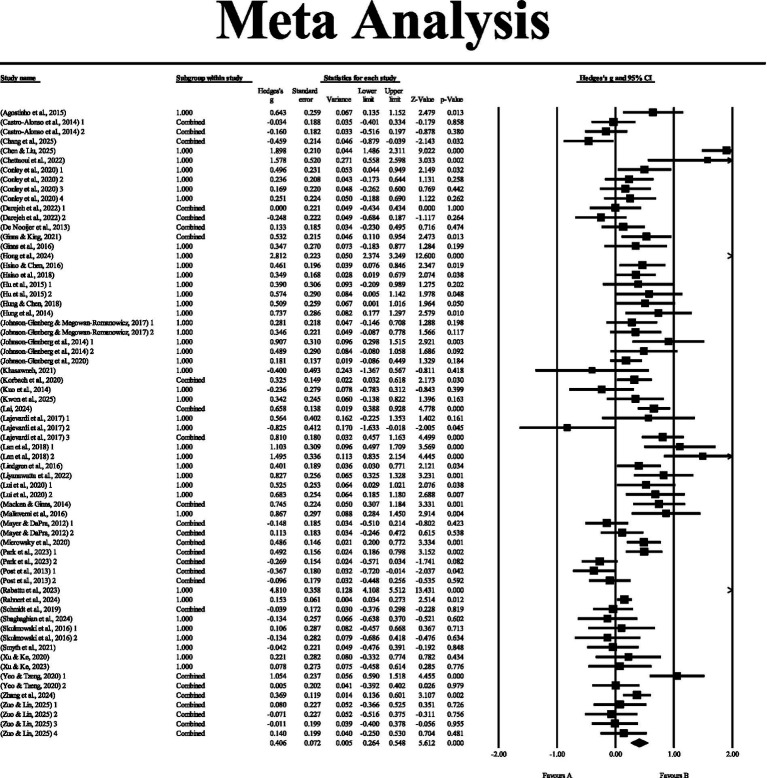
Forest plot.

As shown in the forest plot (Figure 2), under the random-effects model, the overall effect size of 46 studies (66 effect sizes) was 0.406, indicating a moderately positive effect of embodied learning on students’ learning performance. The two-tailed Z test was significant [*Z* = 5.612, 95%CI (0.264, 0.548), *p* < 0.001], demonstrating that embodied learning can significantly enhance students’ learning performance.

### 4.4 Moderator analysis

Table 3 presents the results of the moderator analysis. The study used the random-effects model to examine whether the effect size across the analyzed studies varied based on moderators, aiming to identify sources of heterogeneity.

**Table 3:** Moderator analysis.

Moderators	*k*	Effect size	z	95%CI	Q_B_
Disciplines					10.163*
Science	39	0.433	4.849***	[0.258, 0.608]	
Humanities	17	0.514	2.535*	[0.117, 0.912]	
Computer	4	0.026	0.207	[−0.217, 0.268]	
Physical education	1	0.461	2.347*	[0.076, 0.846]	
Others	5	0.131	0.861	[−0.167, 0.428]	
Educational levels					10.050*
University	37	0.245	2.950**	[0.082, 0.408]	
High school	3	0.742	4.546***	[0.422, 1.062]	
Middle school	3	1.185	1.684	[−0.194, 2.563]	
Primary school	21	0.531	3.928***	[0.266, 0.795]	
Pre-school	2	0.396	3.102**	[0.146, 0.647]	
Experiment periods					9.625*
1 h	46	0.218	4.564***	[0.124, 0.312]	
1 week	7	0.681	2.712**	[0.189, 1.174]	
1 month	3	0.741	0.692	[−1.356, 2.837]	
1 term	6	1.522	2.936**	[0.506, 2.538]	
Sample sizes					7.221*
0–30	46	0.259	4.089***	[0.135, 0.383]	
31–50	10	0.374	4.286***	[0.203, 0.545]	
> 50	10	1.088	3.442**	[0.469, 1.707]	
Regions					4.870
Africa	2	0.772	1.093	[−0.612, 2.156]	
Asia	19	0.609	3.588***	[0.276, 0.941]	
Australia	15	0.269	2.507*	[0.059, 0.479]	
Europe	11	0.487	1.837	[−0.032, 1.007]	
North America	19	0.259	4.553***	[0.147, 0.370]	
Learning approaches					8.680*
Small group	11	0.477	6.078***	[0.323, 0.631]	
Individual	53	0.411	4.746***	[0.241, 0.580]	
Mixed	2	−0.275	−1.133	[−0.752, 0.201]	
Embodied levels					7.141*
High	29	0.647	4.419***	[0.360, 0.934]	
Middle	14	0.225	1.936	[−0.003, 0.453]	
Low	23	0.219	2.892**	[0.071, 0.367]	
Embodied types					5.401*
Active	58	0.445	5.315***	[0.281, 0.610]	
Passive	8	0.112	0.968	[−0.115, 0.340]	

k, number of effect size; Q_B_, intergroup homogeneity; CI, confidence interval.

*p < 0.05; **p < 0.01; ***p < 0.001.

For the effect size of each discipline, humanities exhibited a moderate effect [*g* = 0.514, 95%CI (0.117, 0.912), *p* < 0.05], followed by a moderate effect of physical education [*g* = 0.461, 95%CI (0.076, 0.846), *p* < 0.05] and science [*g* = 0.433, 95%CI (0.258, 0.608), *p* < 0.001]. However, the small effect of computers [*g* = 0.026, 95%CI (−0.217, 0.268), *p* > 0.05] did not reach statistical significance. Overall, the intergroup effect was significant (Q_B_ = 10.163, *p* < 0.05), indicating a significant difference in the effect of embodied learning on students’ learning performance across different disciplines.

For the effect size of each educational level, high school showed a large effect [*g* = 0.742, 95%CI (0.422, 1.062), *p* < 0.001], followed by primary school with a moderate effect [*g* = 0.531, 95%CI (0.266, 0.795), *p* < 0.001], and small effect sizes for pre-school [*g* = 0.396, 95%CI (0.146, 0.647), *p* < 0.01] and university [*g* = 0.245, 95%CI (0.082, 0.408), *p* < 0.01]. However, the large effect of middle school [*g* = 1.185, 95%CI (−0.194, 2.563), *p* > 0.05] did not reach statistical significance. Overall, the intergroup effect was significant (Q_B_ = 10.050, *p* < 0.05), indicating a significant difference in the impact of embodied learning on students’ learning performance across different educational levels.

For the effect size of each experiment period, 1 term showed a large effect [*g* = 1.522, 95%CI (0.506, 2.538), *p* < 0.01], followed by 1 week with a moderate effect [*g* = 0.681, 95%CI (0.189, 1.174), *p* < 0.01], and 1 h with a small effect [*g* = 0.218, 95%CI (0.124, 0.312), *p* < 0.001]. However, the large effect for 1 month [*g* = 0.741, 95%CI (−1.356, 2.837), *p* > 0.05] did not reach statistical significance. Overall, the intergroup effect was significant (Q_B_ = 9.625, *p* < 0.01), indicating a significant difference in the effect of embodied learning on students’ learning performance across different experiment periods.

For the effect size of each sample size, >50 participants showed a moderate effect [*g* = 1.088, 95%CI (0.469, 1.707), *p* < 0.01], followed by a small effect of 31–50 [*g* = 0.374, 95%CI (0.203, 0.545), *p* < 0.001] and 0–30 participants [*g* = 0.259, 95%CI (0.135, 0.383), *p* < 0.001]. The intergroup effect was significant (Q_B_ = 7.221, *p* < 0.05), indicating that the improvement in students’ learning performance differ significantly across different sample sizes.

For the effect size of each region, Asia had a moderate effect [*g* = 0.609, 95%CI (0.276, 0.941), *p* < 0.001], followed by a small effect of Australia [*g* = 0.269, 95%CI (0.059, 0.479), *p* < 0.05] and North America [*g* = 0.259, 95%CI (0.147, 0.370), *p* < 0.001]. However, the large effect of Africa [*g* = 0.772, 95%CI (−0.612, 2.156), *p* > 0.05] and the moderate effect of Europe [*g* = 0.487, 95%CI (−0.032, 1.007), *p* > 0.05] did not reach statistical significance. Furthermore, the intergroup effect was not significant (Q_B_ = 4.870, *p* > 0.05), indicating that the impact of embodied learning on students’ learning performance did not vary significantly across different regions.

For the effect size of each learning approach, small group learning had a moderate effect [*g* = 0.477, 95%CI (0.323, 0.631), *p* < 0.001], followed by a moderate effect of individual learning [*g* = 0.411, 95%CI (0.241, 0.580), *p* < 0.001]. However, the small effect of mixed learning [*g* = −0.275, 95%CI (−0.752, 0.201), *p* > 0.05] did not reach statistical significance. Overall, the intergroup effect was significant (Q_B_ = 8.680, *p* < 0.05), indicating a significant difference in the effect of embodied learning on students’ learning performance across different learning approaches.

For the effect size of each embodied level, the moderate effect of high embodiment [*g* = 0.647, 95%CI (0.360, 0.934), *p* < 0.001] was greater than the small effect of low embodiment [*g* = 0.219, 95%CI (0.071, 0.367), *p* < 0.01]. The small effect of middle embodiment [*g* = 0.225, 95%CI (−0.003, 0.453), *p* > 0.05] did not reach statistical significance. However, the intergroup effect was significant (Q_B_ = 7.141, *p* < 0.05), showing that there was a significant difference in the effects of different embodied levels on students’ learning performance.

For the effect size of each embodied type, the moderate effect of active embodiment [*g* = 0.445, 95%CI (0.281, 0.610), *p* < 0.001] was greater than the small effect of passive embodiment [*g* = 0.112, 95%CI (−0.115, 0.340), *p* > 0.05]. Overall, the results of the intergroup test (Q_B_ = 5.401, *p* < 0.05) showed significant differences in the effect of different embodied types on students’ learning performance.

### 4.5 Publication bias and sensitivity analysis

**Figure 3:**
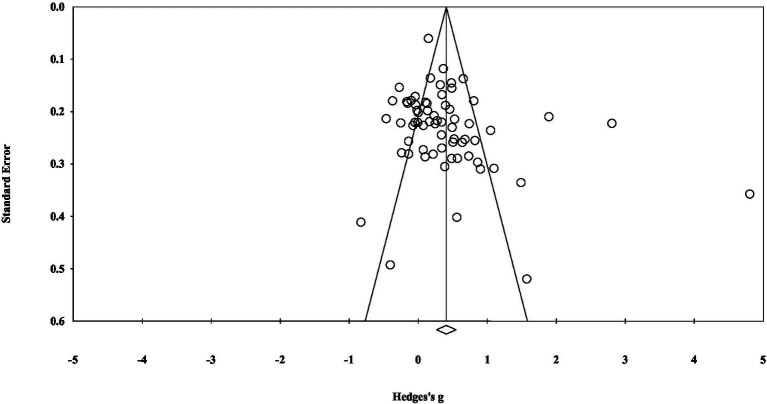
Funnel plot.

The funnel plot (Figure 3) indicated that the effect sizes from most studies were symmetrically distributed around the mean effect size. The trim-and-fill method also suggested that four additional studies would be added to the right side of this funnel plot to achieve symmetry. These findings suggest a low likelihood of publication bias.

Moreover, Orwin’s fail-safe N test (Table 4) indicated that, at the 0.01 significance level, 2,007 unpublished studies would be needed to alter the total effect size. The classic fail-safe N test (Table 5) also indicated that 3,357 unpublished studies would be required to reduce the overall effect size, far exceeding the threshold of 240 studies. Additionally, Egger’s test (*p* = 0.052 > 0.05) did not reach statistical significance. Based on these findings, no evidence of publication bias was found, suggesting that the validity of the data included in this meta-analysis is high.

**Table 4:** Results of Orwin’s fail-safe *N.*

Hedges’ g in observed studies (random effect)	0.31
The criterion for a ‘trivial’ Hedges’ g	0.01
Mean Hedges’ g in missing studies	0.00
Number missing studies needed to bring Hedge’s g under 0.01	2007.00

**Table 5:** Results of classic fail-safe *N.*

*z*-value for observed studies	14.11
*p*-value for observed studies	0.00
Alpha	0.05
Tails	2.00
*z* for alpha	1.96
Number of observed studies	66.00
Number of missing studies that would bring *p*-value to > alpha	3357.00

Finally, the results of sensitivity analysis showed that excluding any study did not affect the meta-analysis outcomes, with all point estimates falling within the range of 95%CI [0.264, 0.548], indicating that the model was robust.

## 5 Discussion

This study employs meta-analysis to examine 46 studies (66 effect sizes) published between 2010 and 2025, exploring the effect of embodied learning on students’ learning performance. Furthermore, it examines the differential effects of moderators, including discipline, educational level, experiment period, sample size, region, learning approach, embodied level, and embodied type.

### 5.1 Embodied learning enhances students’ learning performance

The present study found that embodied learning has a moderate positive effect on students’ learning performance, with an overall effect size of 0.406. This finding aligns with the studies by Kwon et al. (2025) and Chettaoui et al. (2022). Specifically, embodied learning activates learners’ physical experiences, facilitating a seamless connection between real-world information and acquired knowledge (Hong et al., 2024). By externalizing their internal perceptions and thoughts, learners enhance the practical application of their skills. Furthermore, embodied learning integrates knowledge with learners’ perceptions and cognition, transforming abstract concepts into concrete or observable forms, thereby optimizing cognitive load (Macken and Ginns, 2014). This finding is further supported by embodied cognition theory, which highlights the importance of sensory experience and physical presence in the learning process (Wilson, 2002). Embodied learning emphasizes the importance of sensory experiences and physical participation in understanding and retaining knowledge. Through sensory experiences and physical movements, learners can intuitively grasp abstract concepts, thereby reducing the cognitive effort required for learning tasks (Hung and Chen, 2018).

### 5.2 The moderator analysis

This study investigates eight moderators to identify factors that may explain differences in effect sizes among studies. The results found that, in addition to region, differences in discipline, educational level, experiment period, sample size, learning approach, embodied level, and embodied type were key factors influencing the effect of embodied learning on students’ learning performance.

From a disciplinary perspective, embodied learning has a greater impact on students’ learning performance in the humanities than physical education or science, with no significant effect observed in computer science. This finding contradicts the results of Zhang et al. (2025). Embodied cognition theory suggests that embodied learning enhances memory and comprehension through physical engagement and sensory experiences. This effect is especially pronounced in the humanities, which rely heavily on situational memory and the understanding of human experiences. For example, VR technology has been shown to improve language skills by simulating real-world scenarios (Chen and Liu, 2025). Research in cognitive science has also indicated that learning in humanities subjects, such as language acquisition, activates the sensorimotor cortex (Willems and Casasanto, 2011). This suggests that language processing involves not only traditional language centers in the brain but also bodily actions and sensory experiences. In contrast, the knowledge base in the sciences is characterized by experimentation, objectivity, and systematicity. Embodied learning can facilitate the sciences through virtual simulations, offering more opportunities for hands-on practice (Agostinho et al., 2015). However, the disciplinary focus on experimentation and systematic methods may limit the role of embodied learning in science education compared to fields where experiential and sensory learning is more integral. For instance, in science, while virtual simulations provide valuable hands-on practice, they might not engage students’ sensorimotor experiences as effectively as the more intuitive and human-centered learning in the humanities. These findings basically align with the results of existing meta-analysis (Lyu and Deng, 2024). On the other hand, computer science centers on logical thinking and symbolic expression, which requires extensive training in mathematical and scientific reasoning. This emphasis on abstract thought and problem-solving may reduce the impact of embodied learning in this field. As a result, the impact of embodied learning in this field is less evident, and its effect on learning performance is not as pronounced as in other disciplines (Darejeh et al., 2022).

From the perspective of educational levels, the effect of embodied learning on students’ performance varies across educational levels, with the largest impact observed in high school, followed by primary school, and smaller effects seen in pre-school and university. This finding is essentially consistent with existing research (Yu and Yu, 2023). High school students, who often face significant academic pressure, can benefit from embodied learning (e.g., using VR), as it makes learning more engaging and enjoyable, thereby effectively reducing their academic stress. According to Piaget’s theory of cognitive developmental stages, younger students (e.g., pre-school students and primary school students), whose abstract reasoning ability has not yet fully developed, may rely more on multimodal input for information processing (Lefa, 2014). Embodied learning offers concrete visual representations, which not only enhance motivation and attention but also serve as cognitive scaffolding for learning. In contrast, university students, who have developed advanced memory strategies and verbal reasoning skills, may find bodily movements and verbal perception less important for learning tasks (Park et al., 2023). This is because cognitive and abstract thinking typically develop with age (Zou et al., 2025), allowing university students to engage in higher-order cognitive activities such as symbolic thinking and logical reasoning. Therefore, they may be more inclined to rely on abstract thinking for learning rather than physical actions, as confirmed by Zhang et al. (2025). Moreover, Lyu & Deng (2024, p.2) argued that younger students (e.g., pre-school students and primary school students) may struggle with the cognitive load induced by poorly designed embodied learning strategies, leading to increased cognitive stress. This may be because simpler embodied interventions (e.g., pointing, tracing) reduce cognitive load, while highly interactive embodied interventions (e.g., VR, AR technologies) may increase cognitive load, potentially leading to lower learning performance among younger students (e.g., pre-school students and primary school students) with limited cognitive capacity. Therefore, future research should explore the optimal level of embodied interaction across different educational levels, balancing cognitive load and learning performance.

From the perspective of experiment periods, the period of students’ participation in the experiment significantly affects their learning performance, with longer periods yielding better results. This aligns with previous research (Kosmas et al., 2019). Embodied learning, which emphasizes physical engagement, perception, and experience, is based on the idea that learners acquire knowledge through hands-on practice. The longer the period of students’ participation in the experiment, the greater its positive influence on various aspects of learning (e.g., memory consolidation, skill formation; Lai, 2024). However, according to cognitive load theory, if the experiment period is too long, especially when the amount of information exceeds the capacity of working memory, cognitive load increases, thereby reducing learning efficiency (Zou et al., 2025). As demonstrated by Yeo and Tzeng (2020), younger learners, such as those in pre-school and primary school, typically have weaker self-regulation skills than university students. Consequently, longer periods of students’ participation in embodied experiments may lead to greater distractions, increasing external cognitive load, and ultimately reducing learning outcomes. Overall, while extended embodied learning can offer significant benefits for skill development, its period should be carefully adjusted to the learner’s age and cognitive abilities. Striking a balance between period and cognitive load is critical to ensuring that embodied learning becomes an effective tool for advancing education.

From the perspective of sample sizes, the number of participants in the embodied intervention group exceeding 50 has the greatest impact on students’ learning performance, compared to groups with 0–30 and 31–50 participants, with significant intergroup differences. This finding contradicts prior studies (Huang et al., 2022; Kuo et al., 2014). One possible explanation for this discrepancy is the use of AR and VR. These immersive tools allow students participating in embodied interventions to interact with 3D simulations, with task complexity adjusted according to individual progress (Rabattu et al., 2023). Such customization ensures that students who participated in embodied activities, regardless of their prior knowledge, can engage with the material at an appropriate level. By providing personalized, immersive learning experiences, these technologies bridge the gap caused by learner diversity and enhance engagement in larger embodied groups. Furthermore, AR and VR enable educators to effectively manage larger embodied groups by automating the tracking of students’ progress and providing real-time feedback (Hsiao et al., 2018). By reducing teachers’ cognitive load, these tools allow them to focus on higher-level instructional tasks while providing individualized support for each student, thereby improving the overall learning experience. Another reason for the divergence may lie in differences in study design. In earlier research, embodied interventions with larger sample sizes often relied on traditional lecture-based methods, where the benefits of bodily interaction were limited due to low levels of student engagement or insufficient instructional adaptation. In contrast, recent studies have implemented more collaborative and interactive designs in larger-sample contexts (Liyanawatta et al., 2022). For instance, peer-assisted learning, group-based problem-solving, or project-based embodied activities, which may amplify the effects of embodiment by encouraging students to exchange ideas, negotiate meaning, and co-construct knowledge through physical and social interaction. These designs align with the core tenets of embodied cognition, emphasizing not only bodily involvement but also contextual and social embeddedness. Therefore, large-group interventions, when supported by appropriate instructional design, can indeed yield strong learning performance.

From a regional perspective, the impact of embodied learning on students’ learning performance is moderate in Asia, small in Australia and North America, and shows no significant effect in Africa and Europe. Notably, no significant intergroup differences are observed across regions. These findings contrast with previous studies (Lyu and Deng, 2024), which suggested that in regions with abundant educational resources, students are often exposed to diverse embodied activities from an early age. This early familiarity with sensory-based and physically engaging learning may facilitate quicker adaptation, but it could also diminish the novelty effect commonly observed in educational settings with fewer resources. From a cultural context perspective, this divergence in findings may be explained by differences in how education is structured and how embodied learning is integrated into cultural practices. In Australia and North America, students may already be accustomed to a variety of learning tools, including digital and embodied methods. As a result, these students might experience a ceiling effect, where the benefits of new learning methods like embodied learning are less pronounced because they are already familiar with similar techniques (Lajevardi et al., 2017). In contrast, in collectivist cultures (e.g., many parts of Asia), where group-based learning and social interaction are emphasized, embodied learning methods may have a stronger impact, as they align with existing cultural practices of learning through interaction and engagement. Furthermore, in underdeveloped regions such as Africa, where traditional, lecture-based teaching methods are more common, the introduction of embodied learning holds more promise. The novelty effect of embodied learning in these contexts, where such methods are less familiar, tends to have a greater impact (Rahnert et al., 2024). In these regions, the introduction of new, engaging, and sensory-based learning techniques could lead to substantial improvements in students’ learning performance, especially as it breaks from traditional, passive forms of education. These findings underscore the importance of considering both cultural context and regional exposure to embodied learning tools. The varying levels of exposure across different regions and cultural contexts likely play a significant role in shaping how students respond to embodied learning interventions.

From the perspective of learning approaches, collective embodied learning is more effective than individual embodied learning, with significant intergroup differences. These findings support Danish et al. (2020), who suggested that the effectiveness of embodied learning depended on the interaction between individuals and their environment, as well as collaboration within groups. Drawing from activity theory, embodied learning is not only an individual cognitive process but also influenced by group interactions (Engeström, 2014). These interactions collectively create a dynamic activity system, in which learners acquire new cognitive perspectives from others while continuously refining their understanding. In other words, embodied activities serve not only as vehicles for individual cognition but also as valuable resources for collaborative learning (Liyanawatta et al., 2022). In contrast, individual embodied learning may have limitations in terms of cognitive diversity, conflicting perspectives, and the lack of group feedback. Therefore, future research should integrate more dynamic interaction into individual embodied learning to address these limitations. Further work should also explore the impact of different cultural backgrounds and social structures on collective embodied learning, aiming to develop more adaptable instructional designs.

From the perspective of embodied levels, high-level embodied learning has a greater impact on students’ learning performance than low-level embodied learning, which is consistent with the findings of Guo and Goh (2015). According to dual coding theory (Clark and Paivio, 1991), information is stored and retrieved more efficiently when it is encoded simultaneously through both verbal and non-verbal channels (e.g., bodily movements). High-level embodied learning allows learners to engage in full-body participatory simulation within a given scenario (Conley et al., 2020). Through large physical movements and immersive activities, learners embody elements of the scene and interact more deeply with the content. In contrast, low-level embodied learning involves visual input and minimal physical activity, offering a more limited sensory experience (Mayer and DaPra, 2012). This reduced sensory input may hinder deeper information processing. However, previous research suggested that high-level embodied learning may sometimes exceed learners’ working memory capacity, leading to challenges in allocating cognitive resources (Georgiou et al., 2019). One possible reason for this is that learners have varying abilities to manage their cognitive resources. Therefore, when designing embodied learning activities, individual differences should be considered to provide personalized support (Johnson-Glenberg et al., 2020). Students should also effectively allocate their cognitive resources to reduce additional cognitive load.

From the perspective of embodied types, this study found that active embodied learning is more effective in enhancing students’ learning performance than passive embodied learning, which aligns with previous studies (Hung et al., 2014; Xu and Ke, 2020). Active embodied learning (e.g., interacting within a mixed reality simulation) involves not only perceiving information visually or audibly, but also physically engaging with it (Lindgren et al., 2016). This physical interaction facilitates multisensory connections in the brain, creating a richer learning experience. By using their bodies to enact or manipulate concepts, students can integrate information more comprehensively than by passive observation alone. Furthermore, information processing theory explains why active engagement can enhance learning. According to this theory, working memory has a limited capacity, and information that is not adequately rehearsed or processed is prone to being forgotten (Baddeley et al., 2021). In this context, active embodied learning serves as an effective tool for reinforcing the material. Through repetitive bodily movements that simulate the content, students engage with the material in a way that strengthens retention. This dynamic process facilitates the transfer of information from working memory to long-term memory, making the learning experience more lasting and meaningful. However, some previous studies have suggested that active embodied learning (e.g., interacting with Kinect) may have a negative effect on immediate knowledge acquisition (Xu and Ke, 2023). This may be due to the specific design aspects of embodied learning. If active embodied learning involves fixed bodily movements and lacks interactive feedback, its effectiveness may not exceed that of passive embodied learning.

## 6 Implications

The present study has important theoretical and practical implications for education. Theoretically, this meta-analysis expands the existing body of research on embodied learning and learning performance by offering quantitative evidence that embodied learning is an effective method for enhancing students’ learning performance. This study also offers a more comprehensive examination of eight moderators, investigating the impact of embodied learning on students’ learning performance. This expands the scope compared to previous meta-analyses (Lyu and Deng, 2024; Yu and Yu, 2023; Zhang et al., 2025). Furthermore, this study provides a theoretical foundation for the future development of educational policies and the incorporation of embodied learning by educators. Practically, the results of the moderator analysis highlight that embodied learning is not suitable for all learning environments and should not be used arbitrarily. Future work should focus on the following eight points:

(1) In humanities courses, role-playing or simulating scenes allows students to experience specific knowledge, thereby deepening their understanding of learning contexts. In sciences courses, VR technology can facilitate students’ intuitive understanding of abstract concepts, such as vectors, mechanics, and geometry. Through interactive actions like touching and dragging, students can explore the dynamic changes in mathematical functions. In physical education, teachers can integrate physical interaction and sensory experiences into the curriculum. For computer science courses, educators should incorporate embodied programming tasks to help students develop their logical thinking and symbolic expression skills through consistent practice.
(2) Embodied learning should be tailored to students’ cognitive development levels. For pre-school and primary school students, simple and intuitive embodied interventions (e.g., gestures and finger tracking) should be used to enhance learning while reducing cognitive load. For high school and university students, advanced technologies (e.g., VR, motion-sensing technology) should be integrated to increase their engagement in embodied activities.
(3) The duration of students’ participation in the experiment should be adjusted according to their age and cognitive abilities. Teachers should balance the duration of students’ participation in the experiment with their cognitive abilities to optimize learning outcomes. Additionally, incorporating breaks and varying activities can help maintain engagement and reduce fatigue, and ensure that embodied learning remains effective across all age groups.
(4) Educators should consider integrating AR and VR technologies into their teaching to enhance personalized learning, especially in larger embodied intervention groups. These tools can manage students who participated in embodied activities by offering tailored learning experiences and reducing cognitive load for teachers, thereby realizing more effective instruction and individualized support.
(5) It is important to consider the educational traditions and learning styles across different regions. In regions with limited educational resources, the focus should be on experimental and inquiry-based learning. In resource-rich regions, embodied activities should focus on engaging students through advanced interactive modes, interdisciplinary integration, and gamified learning to capture their attention.
(6) Teachers should effectively integrate individual practice with collaborative activities, enabling students to engage in independent thinking while expanding their knowledge through group interactions. More embodied activities based on group collaboration should be designed into the learning process, such as open classrooms and interactive laboratories.
(7) Teachers should consider the cognitive load and adjust the balance between high and low embodied learning based on the nature of the learning tasks. By carefully matching the type of embodied learning with different stages of the learning process, educators can help students effectively manage their cognitive resources. Additionally, personalized support should be provided based on individual differences, ensuring that students are not overwhelmed and can focus on learning in a way that aligns with their cognitive abilities.
(8) The combination of active and passive embodied learning can create a dynamic learning environment that accommodates diverse learning preferences. By integrating real-time feedback into active embodied activities and incorporating meaningful observational tasks into passive embodied learning, teachers can offer a comprehensive approach that fosters deeper understanding.

## 7 Limitations and future direction

This study has several limitations. First, some moderators have small sample sizes, which restricts the study of interactions between moderators. Future work should expand sample sizes to further investigate the interactions effect of moderators, such as those between educational level and experiment period. Second, due to the researchers’ language limitations, non-English studies were excluded from this meta-analysis. While this decision was made to ensure accurate data extraction and interpretation, it inevitably introduces a potential language bias. Excluding non-English studies may limit the generalizability of the findings, particularly in non-English-speaking contexts where cultural and educational practices related to embodied learning might differ significantly. This bias may also lead to an underrepresentation of regional perspectives and outcomes. Future research should aim to include studies published in other languages to provide a more comprehensive understanding of how embodied learning affects learners across diverse linguistic and cultural backgrounds. Finally, this study included only (quasi-)experimental studies with available data for meta-analysis, excluding relevant qualitative research. Future work should combine qualitative analysis with meta-analysis to conduct a more comprehensive analysis.

## 8 Conclusion

This study employs a meta-analysis to examine the effect of embodied learning on students’ learning performance. By analyzing 46 studies and calculating 66 effect sizes, the results reveal a moderately positive impact of embodied learning on students’ learning performance. Further analysis of eight moderators indicates that embodied learning has no significant effect on students’ learning performance across different regions, but significant differences are observed in different disciplines, educational levels, experiment periods, sample sizes, learning approaches, embodied levels, and embodied types. In conclusion, the findings suggest that the positive effect of embodied learning on students’ learning performance and its effect varies under different learning contexts and application conditions. Based on these findings, educators can incorporate embodied activities into their teaching and encourage students to apply embodied learning appropriately.

## Data Availability

The original contributions presented in the study are included in the article/Supplementary material, further inquiries can be directed to the corresponding author/s.

## Notes

References marked with an asterisk indicate studies included in the meta‑analysis.
