# Supplementary material for: The effect of embodied learning on students’ learning performance: A meta-analysis
Source: Front Psychol. 2025 Aug 22;16:1658797. doi: 10.3389/fpsyg.2025.1658797 (PMC12411960; doi:10.3389/fpsyg.2025.1658797)
Supplement: Supplementary file 1 [file Supplementary_file_1.docx]

| Study names | Subgroup | Treatment | | | Control | | | Disciplines | Educational levels | Experiment periods | Sample sizes | Regions | Learning approaches | Embodied levels | Embodied types |
| --- | --- | --- | --- | --- | --- | --- | --- | --- | --- | --- | --- | --- | --- | --- | --- |
|  |  | M | SD | Sample | M | SD | Sample |  |  |  |  |  |  |  |  |
| (Xu & Ke, 2023) | 1 | 5.84 | 2.99 | 25 | 5.63 | 2.31 | 27 | Computer | University | 1 h | 0-30 | North America | Individual | High | Active |
| (Zuo & Lin, 2025) 1 | 1 | 23.79 | 6.05 | 19 | 22.95 | 3.78 | 19 | Science | University | 1 h | 0-30 | Asia | Individual | Low | Active |
| (Zuo & Lin, 2025) 1 | 2 | 7 | 3.27 | 19 | 6.32 | 3.09 | 19 | Science | University | 1 h | 0-30 | Asia | Individual | Low | Active |
| (Zuo & Lin, 2025) 2 | 1 | 21.37 | 5.94 | 19 | 22.95 | 3.78 | 19 | Science | University | 1 h | 0-30 | Asia | Individual | High | Active |
| (Zuo & Lin, 2025) 2 | 2 | 6.63 | 2.39 | 19 | 6.32 | 3.09 | 19 | Science | University | 1 h | 0-30 | Asia | Individual | High | Active |
| (Zuo & Lin, 2025) 3 | 1 | 15.79 | 4.17 | 24 | 15.81 | 4.27 | 26 | Science | University | 1 h | 0-30 | Asia | Individual | Low | Active |
| (Zuo & Lin, 2025) 3 | 2 | 10.33 | 4.06 | 24 | 10.42 | 3.78 | 26 | Science | University | 1 h | 0-30 | Asia | Individual | Low | Active |
| (Zuo & Lin, 2025) 4 | 1 | 16.13 | 4.37 | 24 | 15.81 | 4.27 | 26 | Science | University | 1 h | 0-30 | Asia | Individual | High | Active |
| (Zuo & Lin, 2025) 4 | 2 | 11.42 | 3.27 | 24 | 10.42 | 3.78 | 26 | Science | University | 1 h | 0-30 | Asia | Individual | High | Active |
| (Xu & Ke, 2020) | 1 | 9.29 | 1.23 | 24 | 8.96 | 1.67 | 25 | Humanities | University | 1 h | 0-30 | North America | Individual | High | Active |
| (Mayer & DaPra, 2012) 1 | 1 | 2.6 | 1.6 | 29 | 3.1 | 1.6 | 29 | Science | University | 1 h | 0-30 | North America | Individual | Low | Passive |
| (Mayer & DaPra, 2012) 1 | 2 | 9.2 | 4.6 | 29 | 10.2 | 5.3 | 29 | Science | University | 1 h | 0-30 | North America | Individual | Low | Passive |
| (Mayer & DaPra, 2012) 2 | 1 | 4.3 | 2.1 | 30 | 3.1 | 1.6 | 29 | Science | University | 1 h | 0-30 | North America | Individual | Low | Passive |
| (Mayer & DaPra, 2012) 2 | 2 | 10.2 | 5.9 | 30 | 10.2 | 5.3 | 29 | Science | University | 1 h | 0-30 | North America | Individual | Low | Passive |
| (Liyanawatta et al., 2022) | 1 | 72.58 | 12.18 | 31 | 62.26 | 12.47 | 34 | Humanities | High school | No report | 31-50 | Asia | Small group | High | Active |
| (Chettaoui et al., 2022) | 1 | 7.33 | 1 | 9 | 5.5 | 1.2 | 9 | Science | Primary school | 1 h | 0-30 | Africa | Individual | High | Active |
| (Darejeh et al., 2022) 1 | 1 | 8.15 | 0.933 | 20 | 8.3 | 0.801 | 20 | Computer | University | No report | 0-30 | Australia | Individual | Low | Active |
| (Darejeh et al., 2022) 1 | 2 | 7.4 | 1.93 | 20 | 7.25 | 1.65 | 20 | Computer | University | No report | 0-30 | Australia | Individual | Low | Active |
| (Darejeh et al., 2022) 2 | 1 | 7.8 | 0.951 | 20 | 8.05 | 0.887 | 20 | Computer | University | No report | 0-30 | Australia | Individual | Low | Passive |
| (Darejeh et al., 2022) 2 | 2 | 6.875 | 1.776 | 20 | 7.325 | 1.558 | 20 | Computer | University | No report | 0-30 | Australia | Individual | Low | Passive |
| (Lan et al., 2018) 1 | 1 | 22.56 | 3.86 | 25 | 18.09 | 4.12 | 22 | Humanities | Primary school | 1 term | 0-30 | Asia | Individual | High | Active |
| (Lan et al., 2018) 2 | 1 | 24.36 | 4.12 | 22 | 18.09 | 4.12 | 22 | Humanities | Primary school | 1 term | 0-30 | Asia | Individual | Low | Passive |
| (Ginns & King, 2021) | 1 | 6.55 | 2.28 | 22 | 4.64 | 2.54 | 22 | Science | University | 1 h | 0-30 | Australia | Individual | Low | Active |
| (Ginns & King, 2021) | 2 | 2.5 | 1.01 | 22 | 1.64 | 1.14 | 22 | Science | University | 1 h | 0-30 | Australia | Individual | Low | Active |
| (Hung et al., 2014) | 1 | 62.48 | 11.383 | 27 | 53.54 | 12.549 | 24 | Science | Primary school | 1 h | 0-30 | Asia | Individual | Middle | Active |
| (Kuo et al., 2014) | 1 | 81.17 | 18.03 | 25 | 85.35 | 16.89 | 25 | Humanities | Primary school | 1 h | 0-30 | Asia | Mixed | High | Active |
| (Agostinho et al., 2015) | 1 | 9.07 | 2.42 | 30 | 7.5 | 2.4 | 31 | Science | Primary school | 1 h | 0-30 | Australia | Individual | Low | Active |
| (Yeo & Tzeng, 2020) 1 | 1 | 1.9 | 0.31 | 20 | 1.65 | 0.59 | 20 | Science | Primary school | 1 h | 0-30 | Asia | Individual | Low | Active |
| (Yeo & Tzeng, 2020) 1 | 2 | 3.2 | 0.77 | 20 | 1.7 | 0.92 | 20 | Science | Primary school | 1 h | 0-30 | Asia | Individual | Low | Active |
| (Yeo & Tzeng, 2020) 2 | 1 | 1.88 | 0.34 | 24 | 1.83 | 0.38 | 24 | Science | Primary school | 1 h | 0-30 | Asia | Individual | Low | Active |
| (Yeo & Tzeng, 2020) 2 | 2 | 2.79 | 1.06 | 24 | 2.83 | 1.09 | 24 | Science | Primary school | 1 h | 0-30 | Asia | Individual | Low | Active |
| (Ginns et al., 2016) | 1 | 5.89 | 2.78 | 27 | 4.93 | 2.67 | 27 | Science | Primary school | 1 h | 0-30 | Australia | Individual | Low | Active |
| (Hu et al., 2015) 1 | 1 | 1.38 | 0.86 | 21 | 1.05 | 0.8 | 21 | Science | Primary school | 1 h | 0-30 | Australia | Individual | Low | Active |
| (Hu et al., 2015) 2 | 1 | 1.67 | 0.7 | 24 | 1.25 | 0.74 | 24 | Science | Primary school | 1 h | 0-30 | Australia | Individual | Low | Active |
| (Park et al., 2023) 1 | 1 | 86.42 | 19.36 | 27 | 79.6 | 21.77 | 29 | Science | University | 1 h | 0-30 | Europe | Individual | Low | Active |
| (Park et al., 2023) 1 | 2 | 80.56 | 18.78 | 27 | 71.78 | 20.84 | 29 | Science | University | 1 h | 0-30 | Europe | Individual | Low | Active |
| (Park et al., 2023) 1 | 3 | 82.34 | 17.57 | 27 | 67.64 | 22.65 | 29 | Science | University | 1 h | 0-30 | Europe | Individual | Low | Active |
| (Park et al., 2023) 2 | 1 | 76.54 | 24.02 | 27 | 79.6 | 21.77 | 29 | Science | University | 1 h | 0-30 | Europe | Individual | Low | Active |
| (Park et al., 2023) 2 | 2 | 64.35 | 26.96 | 27 | 71.78 | 20.84 | 29 | Science | University | 1 h | 0-30 | Europe | Individual | Low | Active |
| (Park et al., 2023) 2 | 3 | 57.83 | 30.12 | 27 | 67.64 | 22.65 | 29 | Science | University | 1 h | 0-30 | Europe | Individual | Low | Active |
| (Korbach et al., 2020) | 1 | 75.21 | 19.1 | 30 | 71.16 | 23.31 | 30 | Science | University | 1 h | 0-30 | Europe | Individual | Low | Active |
| (Korbach et al., 2020) | 2 | 89.38 | 14.14 | 30 | 78.48 | 25.65 | 30 | Science | University | 1 h | 0-30 | Europe | Individual | Low | Active |
| (Korbach et al., 2020) | 3 | 74.36 | 17.33 | 30 | 67.76 | 26.51 | 30 | Science | University | 1 h | 0-30 | Europe | Individual | Low | Active |
| (Lindgren et al., 2016) | 1 | 4.84 | 1.78 | 58 | 4.13 | 1.74 | 55 | Science | Middle school | 1 h | >51 | North America | Individual | High | Active |
| (Skulmowski et al., 2016) 1 | 1 | 6 | 2.64 | 24 | 5.7 | 2.95 | 23 | Science | University | 1 h | 0-30 | Europe | Individual | Middle | Active |
| (Skulmowski et al., 2016) 2 | 1 | 5.62 | 2.57 | 24 | 5.96 | 2.42 | 25 | Science | University | 1 h | 0-30 | Europe | Individual | Middle | Active |
| (Smyth et al., 2021) | 1 | 49.23 | 18.1 | 51 | 49.93 | 13.07 | 33 | Science | University | 1 h | >51 | Australia | Small group | Middle | Active |
| (Hung & Chen, 2018) | 1 | 55.33 | 10.42 | 30 | 50 | 10.26 | 30 | Science | University | 1 h | 0-30 | Asia | Individual | Middle | Active |
| (Macken & Ginns, 2014) | 1 | 15.9 | 2.41 | 21 | 12.62 | 4.65 | 21 | Science | University | 1 h | 0-30 | Australia | Individual | Low | Active |
| (Macken & Ginns, 2014) | 2 | 13.95 | 2.6 | 21 | 11.86 | 3.97 | 21 | Science | University | 1 h | 0-30 | Australia | Individual | Low | Active |
| (Lai, 2024) | 1 | 1.8 | 0.405 | 40 | 1.12 | 0.64 | 34 | Others | Primary school | 1 term | 31-50 | Asia | Small group | High | Active |
| (Lai, 2024) | 2 | 1.7 | 0.464 | 40 | 1.06 | 0.6 | 34 | Others | Primary school | 1 term | 31-50 | Asia | Small group | High | Active |
| (Lai, 2024) | 3 | 4.73 | 1.261 | 40 | 3.09 | 1.357 | 34 | Others | Primary school | 1 term | 31-50 | Asia | Small group | High | Active |
| (Hsiao et al., 2018) | 1 | 4.17 | 1.34 | 70 | 3.68 | 1.45 | 72 | Science | Pre-school | 1 h | >51 | Asia | Individual | High | Active |
| (Post et al., 2013) 1 | 1 | 0.58 | 0.29 | 16 | 0.76 | 0.19 | 15 | Humanities | Primary school | 1 h | 0-30 | Europe | Individual | Middle | Active |
| (Post et al., 2013) 1 | 2 | 0.45 | 0.35 | 16 | 0.58 | 0.37 | 15 | Humanities | Primary school | 1 h | 0-30 | Europe | Individual | Middle | Active |
| (Post et al., 2013) 1 | 3 | 0.32 | 0.24 | 16 | 0.41 | 0.26 | 15 | Humanities | Primary school | 1 h | 0-30 | Europe | Individual | Middle | Active |
| (Post et al., 2013) 1 | 4 | 0.41 | 0.41 | 16 | 0.5 | 0.38 | 15 | Humanities | Primary school | 1 h | 0-30 | Europe | Individual | Middle | Active |
| (Post et al., 2013) 2 | 1 | 0.85 | 0.18 | 17 | 0.9 | 0.17 | 14 | Humanities | Primary school | 1 h | 0-30 | Europe | Individual | Middle | Active |
| (Post et al., 2013) 2 | 2 | 0.7 | 0.38 | 17 | 0.76 | 0.38 | 14 | Humanities | Primary school | 1 h | 0-30 | Europe | Individual | Middle | Active |
| (Post et al., 2013) 2 | 3 | 0.59 | 0.26 | 17 | 0.54 | 0.17 | 14 | Humanities | Primary school | 1 h | 0-30 | Europe | Individual | Middle | Active |
| (Post et al., 2013) 2 | 4 | 0.65 | 0.4 | 17 | 0.71 | 0.29 | 14 | Humanities | Primary school | 1 h | 0-30 | Europe | Individual | Middle | Active |
| (Chang et al., 2025) | 1 | -0.13 | 0.17 | 22 | 0.13 | 0.24 | 22 | Humanities | University | 1 month | 0-30 | Asia | Individual | Low | Active |
| (Chang et al., 2025) | 2 | 0 | 0.31 | 22 | 0 | 0.35 | 22 | Humanities | University | 1 month | 0-30 | Asia | Individual | Low | Active |
| (Rabattu et al., 2023) | 1 | 11.58 | 0.55 | 60 | 8.99 | 0.52 | 61 | Science | University | 1 term | >51 | Europe | Individual | High | Active |
| (Conley et al., 2020) 1 | 1 | 1.44 | 1.9 | 36 | 0.58 | 1.53 | 40 | Science | University | 1 h | 31-50 | North America | Small group | High | Active |
| (Conley et al., 2020) 2 | 1 | 0.98 | 1.79 | 53 | 0.58 | 1.53 | 40 | Science | University | 1 h | >51 | North America | Small group | Middle | Active |
| (Conley et al., 2020) 3 | 1 | 0.98 | 2.41 | 44 | 0.61 | 1.85 | 38 | Science | University | 1 h | 31-50 | North America | Individual | High | Active |
| (Conley et al., 2020) 4 | 1 | 1.07 | 1.78 | 41 | 0.61 | 1.85 | 38 | Science | University | 1 h | 31-50 | North America | Individual | Middle | Active |
| (Lui et al., 2020) 1 | 1 | 83.85 | 18.05 | 17 | 71.23 | 24.36 | 200 | Science | University | 1 term | 0-30 | North America | Individual | High | Active |
| (Lui et al., 2020) 2 | 1 | 87.5 | 14.22 | 17 | 71.23 | 24.36 | 200 | Science | University | 1 term | 0-30 | North America | Individual | Middle | Active |
| (Castro-Alonso et al., 2014) 1 | 1 | 6.04 | 2.78 | 28 | 6.14 | 2.05 | 28 | Others | University | 1 h | 0-30 | Australia | Individual | Low | Passive |
| (Castro-Alonso et al., 2014) 1 | 2 | 7.46 | 2.77 | 28 | 7.54 | 2.69 | 28 | Others | University | 1 h | 0-30 | Australia | Individual | Low | Passive |
| (Castro-Alonso et al., 2014) 2 | 1 | 4.67 | 2.02 | 30 | 5.13 | 2.57 | 30 | Others | University | 1 h | 0-30 | Australia | Individual | Low | Passive |
| (Castro-Alonso et al., 2014) 2 | 2 | 6.6 | 3.11 | 30 | 7.03 | 2.66 | 30 | Others | University | 1 h | 0-30 | Australia | Individual | Low | Passive |
| (Shaghaghian et al., 2024) | 1 | 71.35 | 20.44 | 29 | 74.36 | 23.67 | 30 | Science | University | 1 month | 0-30 | North America | Individual | High | Active |
| (Johnson-Glenberg et al., 2014b) 1 | 1 | 60.62 | 32.65 | 16 | 38.89 | 18.23 | 35 | Science | High school | 1 week | 0-30 | North America | Small group | High | Active |
| (Johnson-Glenberg et al., 2014b) 2 | 1 | 47.19 | 14.46 | 39 | 40.17 | 13.37 | 17 | Science | High school | 1 week | 31-50 | North America | Small group | High | Active |
| (Johnson-Glenberg et al., 2020) | 1 | 18.47 | 4.86 | 105 | 17.47 | 6.04 | 109 | Science | University | 1 h | >51 | North America | Individual | High | Active |
| (Johnson-Glenberg & Megowan-Romanowicz, 2017) 1 | 1 | 48.5 | 12.1 | 45 | 44.8 | 14.1 | 39 | Science | University | 1 h | 31-50 | North America | Individual | Low | Passive |
| (Johnson-Glenberg & Megowan-Romanowicz, 2017) 2 | 1 | 49.3 | 11.7 | 43 | 44.8 | 14.1 | 39 | Science | University | 1 h | 31-50 | North America | Individual | High | Active |
| (Hong et al., 2024) | 1 | 7.99 | 0.56 | 82 | 6.63 | 0.38 | 77 | Humanities | Middle school | 1 month | >51 | Asia | Individual | High | Active |
| (Schmidt et al., 2019) | 1 | 4.62 | 2.47 | 34 | 2.27 | 1.63 | 33 | Humanities | Primary school | 1 week | 31-50 | Europe | Individual | High | Active |
| (Schmidt et al., 2019) | 2 | 107.17 | 12.39 | 34 | 113.83 | 11.76 | 33 | Humanities | Primary school | 1 week | 31-50 | Europe | Individual | High | Active |
| (Lajevardi et al., 2017) 1 | 1 | 8.41 | 0.51 | 12 | 8.1 | 0.55 | 12 | Humanities | University | 1 h | 0-30 | Australia | Individual | Middle | Active |
| (Lajevardi et al., 2017) 2 | 1 | 7.9 | 0.39 | 12 | 8.26 | 0.45 | 12 | Humanities | University | 1 h | 0-30 | Australia | Individual | Middle | Active |
| (Lajevardi et al., 2017) 3 | 1 | 0.95 | 0.07 | 22 | 0.75 | 0.21 | 22 | Humanities | Primary school | 1 h | 0-30 | Australia | Individual | Middle | Active |
| (Lajevardi et al., 2017) 3 | 2 | 0.9 | 0.15 | 22 | 0.73 | 0.25 | 22 | Humanities | Primary school | 1 h | 0-30 | Australia | Individual | Middle | Active |
| (Lajevardi et al., 2017) 3 | 3 | 0.76 | 0.23 | 22 | 0.55 | 0.34 | 22 | Humanities | Primary school | 1 h | 0-30 | Australia | Individual | Middle | Active |
| (De Nooijer et al., 2013) | 1 | 50 | 23 | 27 | 45 | 25 | 31 | Humanities | Primary school | 1 h | 0-30 | Europe | Individual | High | Active |
| (De Nooijer et al., 2013) | 2 | 35 | 28 | 27 | 33 | 26 | 31 | Humanities | Primary school | 1 h | 0-30 | Europe | Individual | High | Active |
| (Mierowsky et al., 2020) | 1 | 86.9 | 19.9 | 24 | 78.4 | 29.7 | 24 | Humanities | University | No report | 0-30 | Australia | Individual | Middle | Active |
| (Mierowsky et al., 2020) | 2 | 86.5 | 21.3 | 24 | 63.1 | 25.1 | 24 | Humanities | University | No report | 0-30 | Australia | Individual | Middle | Active |
| (Mierowsky et al., 2020) | 3 | 78.4 | 20.4 | 24 | 59.9 | 30.7 | 24 | Humanities | University | No report | 0-30 | Australia | Individual | Middle | Active |
| (Mierowsky et al., 2020) | 4 | 52.3 | 37 | 24 | 44.3 | 29.2 | 24 | Humanities | University | No report | 0-30 | Australia | Individual | Middle | Active |
| (Hsiao & Chen, 2016) | 1 | 13.73 | 4.31 | 52 | 11.4 | 5.63 | 53 | Physical education | Pre-school | 1 h | >51 | Asia | Small group | High | Active |
| (Johnson-Glenberg et al., 2014a) | 1 | 73.5 | 10.51 | 10 | 78.05 | 11.19 | 6 | Others | Primary school | 1 h | 0-30 | North America | Mixed | High | Active |
| (Malinverni et al., 2016) | 1 | 9.14 | 2.88 | 24 | 6.62 | 2.84 | 24 | Science | Primary school | 1 week | 0-30 | Europe | Small group | High | Active |
| (Rahnert et al., 2024) | 1 | 13.06 | 2.94 | 571 | 12.6 | 3.06 | 509 | Others | University | 1 h | >51 | Africa | Individual | Low | Passive |
| (Kwon et al., 2025) | 1 | 3.3 | 1.759 | 33 | 2.73 | 1.526 | 33 | Computer | Primary school | 1 week | 31-50 | North America | Small group | High | Active |
| (Chen & Liu, 2025) | 1 | 132.7 | 13.41 | 64 | 103.7 | 16.73 | 66 | Humanities | Primary school | 1 week | >51 | Asia | Individual | High | Active |
| (Zhang et al., 2024) | 1 | 3.31 | 1.57 | 25 | 2.94 | 1.24 | 23 | Humanities | Middle school | 1 week | 0-30 | Asia | Small group | High | Active |
| (Zhang et al., 2024) | 2 | 5.07 | 0.89 | 25 | 4.56 | 1.11 | 23 | Humanities | Middle school | 1 week | 0-30 | Asia | Small group | High | Active |
| (Zhang et al., 2024) | 3 | 6.01 | 0.78 | 25 | 5.47 | 1.1 | 23 | Humanities | Middle school | 1 week | 0-30 | Asia | Small group | High | Active |
| (Zhang et al., 2024) | 4 | 5.54 | 0.86 | 25 | 5.11 | 1.1 | 23 | Humanities | Middle school | 1 week | 0-30 | Asia | Small group | High | Active |
| (Zhang et al., 2024) | 5 | 5.25 | 0.81 | 25 | 4.59 | 1.08 | 23 | Humanities | Middle school | 1 week | 0-30 | Asia | Small group | High | Active |
| (Zhang et al., 2024) | 6 | 5.34 | 0.85 | 25 | 4.89 | 1.16 | 23 | Humanities | Middle school | 1 week | 0-30 | Asia | Small group | High | Active |
